# Genomic Characterization of *Klebsiella pneumoniae* Causing Invasive Disease in South African Infants: Observational Studies Between 2018 and 2023

**DOI:** 10.1093/ofid/ofag004

**Published:** 2026-01-20

**Authors:** Courtney P Olwagen, Alane Izu, Shama Khan, Stephanie Jones, Carmen Briner, Gaurav Kwatra, Lara Van der Merwe, Nicholas J Dean, Vicky L Baillie, Sana Mahtab, Kimberleigh Storath, Imaan Dunn, Lubomira Andrew, Urvi Rajyaguru, Firdose L Nakwa, Sithembiso C Velaphi, Jeannette Wadula, Renate Strehlau, Anika M van Niekerk, Niree Naidoo, Yogandree Ramsamy, Mohamed Said, Robert G K Donald, Raphael Simon, Ziyaad Dangor, Shabir A Madhi

**Affiliations:** South African Medical Research Council Vaccines and Infectious Diseases Analytics Research Unit, Faculty of Health Science, University of the Witwatersrand, Johannesburg, South Africa; South African Medical Research Council Vaccines and Infectious Diseases Analytics Research Unit, Faculty of Health Science, University of the Witwatersrand, Johannesburg, South Africa; South African Medical Research Council Vaccines and Infectious Diseases Analytics Research Unit, Faculty of Health Science, University of the Witwatersrand, Johannesburg, South Africa; South African Medical Research Council Vaccines and Infectious Diseases Analytics Research Unit, Faculty of Health Science, University of the Witwatersrand, Johannesburg, South Africa; South African Medical Research Council Vaccines and Infectious Diseases Analytics Research Unit, Faculty of Health Science, University of the Witwatersrand, Johannesburg, South Africa; South African Medical Research Council Vaccines and Infectious Diseases Analytics Research Unit, Faculty of Health Science, University of the Witwatersrand, Johannesburg, South Africa; Division of Infectious Diseases, Department of Pediatrics, Cincinnati Children's Hospital Medical Center and University of Cincinnati, Cincinnati, Ohio, USA; Department of Clinical Microbiology, Christian Medical College, Vellore, India; South African Medical Research Council Vaccines and Infectious Diseases Analytics Research Unit, Faculty of Health Science, University of the Witwatersrand, Johannesburg, South Africa; South African Medical Research Council Vaccines and Infectious Diseases Analytics Research Unit, Faculty of Health Science, University of the Witwatersrand, Johannesburg, South Africa; South African Medical Research Council Vaccines and Infectious Diseases Analytics Research Unit, Faculty of Health Science, University of the Witwatersrand, Johannesburg, South Africa; South African Medical Research Council Vaccines and Infectious Diseases Analytics Research Unit, Faculty of Health Science, University of the Witwatersrand, Johannesburg, South Africa; South African Medical Research Council Vaccines and Infectious Diseases Analytics Research Unit, Faculty of Health Science, University of the Witwatersrand, Johannesburg, South Africa; South African Medical Research Council Vaccines and Infectious Diseases Analytics Research Unit, Faculty of Health Science, University of the Witwatersrand, Johannesburg, South Africa; Vaccine Research and Development, Pfizer, Pearl River, New York, USA; Vaccine Research and Development, Pfizer, Pearl River, New York, USA; Department of Paediatrics and Child Health, Chris Hani Baragwanath Academic Hospital, School of Clinical Medicine, Faculty of Health Sciences, University of the Witwatersrand, Johannesburg, South Africa; Department of Paediatrics and Child Health, Chris Hani Baragwanath Academic Hospital, School of Clinical Medicine, Faculty of Health Sciences, University of the Witwatersrand, Johannesburg, South Africa; Department of Clinical Microbiology and Infectious Diseases, National Health Laboratory Services, Faculty of Health Science, University of Witwatersrand, Johannesburg, South Africa; Wits VIDA Nkanyezi Research Unit, Department of Paediatrics and Child Health, School of Clinical Medicine, University of the Witwatersrand, Johannesburg, South Africa; Mowbray Maternity Hospital, Division of Neonatal Medicine, Department of Paediatrics, Faculty of Health Sciences, University of Cape Town, Town, South Africa; Department of Clinical Microbiology and Infectious Diseases, National Health Laboratory Services, Faculty of Health Science, University of Witwatersrand, Johannesburg, South Africa; Antimicrobial Research Unit, University of KwaZulu-Natal, KwaZulu-Natal South Africa; Department of Medical Microbiology, National Health Laboratory Service, Prince Mshiyeni Hospital, KwaZulu-Natal, South Africa; Department of Medical Microbiology, University of Pretoria, Pretoria, South Africa; National Health Laboratory Services, Tshwane Academic Division, Tshwane, South Africa; Vaccine Research and Development, Pfizer, Pearl River, New York, USA; Vaccine Research and Development, Pfizer, Pearl River, New York, USA; South African Medical Research Council Vaccines and Infectious Diseases Analytics Research Unit, Faculty of Health Science, University of the Witwatersrand, Johannesburg, South Africa; South African Medical Research Council Vaccines and Infectious Diseases Analytics Research Unit, Faculty of Health Science, University of the Witwatersrand, Johannesburg, South Africa; Wits Infectious Diseases and Oncology Research Institute, Faculty of Health Science, University of the Witwatersrand, Johannesburg, South Africa

**Keywords:** *Klebsiella pneumoniae*-associated deaths, multidrug resistance, neonatal sepsis, serious bacterial infection, vaccine

## Abstract

**Background:**

*Klebsiella pneumoniae* (KPn) is a leading cause of invasive bacterial disease in African children, albeit with a scarcity of genotypic characterization.

**Methods:**

We sequenced invasive KPn isolates from infants ≤90 days, collected through observational hospital surveillance (n = 226) between March 4, 2019 and February 27, 2021, and between May 13, 2022 and October 31, 2023, and postmortem sampling (n = 111) between February 15, 2018 and April 18, 2023. Postmortem Kpn isolates were attributed in the causal pathway to death by the determination of the cause of death panel, which consists of local experts.

**Results:**

Three hundred and thirty-seven isolates (226 identified during hospital surveillance and 111 from postmortem sampling) were included in the final analysis. Genomic analysis identified 85 distinct clonotypes. Sequence type (ST) 17 (22.0%) predominated, followed by ST39 (12.7%). The dominant K-locus (KL) were KL25 (24.0%), KL2 (14.5%), and KL149 (13/4%), while the dominant O-antigens included O1αβ,2α(48.4%), and O5 (19.9%). Eighty-five percent (287/337) of the KPn isolates harbored multidrug resistant genes, including 32.9% to carbapenems. Notably, bla_OXA-181_, bla_NDM-5_, and bla_NDM-1_ were detected in 26.4%, 2.1% (7/337), and 0.3% (1/337) of isolates, respectively.

**Conclusions:**

Although a wide diversity of strains were associated with Kpn invasive disease, over 80% of the cases were attributed to 11 K loci. These data provide critical insights into KPn epidemiology and highlight potential antigen targets for vaccine development in young African children.

## BACKGROUND

Sepsis is a leading cause of death in young infants (≤90 days of age) in low-middle-income countries (LMIC) [1]. Global estimates indicate that the incidence of sepsis among young infants ranges from 1.3 to 3.9 million cases, with sepsis-related complications leading to approximately 400 000 to 700 000 deaths each year [2], 42% of which occur within the first week of life [3]. The burden of invasive bacterial disease is likely underestimated in LMIC, due to limited access to health facilities and laboratory resources to determine the cause of sepsis [4]. In sub-Saharan Africa, the reported incidence of neonatal sepsis ranges from 4.5 to 21 per 1000 live births, with a case-fatality risk of 27% to 56% [4]. In contrast, the estimated incidence of neonatal sepsis is 0.8 to 1 per 1000 in the United States, where the case-fatality risk is 3% to 19% [5, 6]. Moreover, there are an estimated 2 million annual stillbirths globally, >98% of which occur in LMIC [7].

Despite the prominent role of *Klebsiella pneumoniae* (KPn) in causing infant sepsis and death, most studies from LMICs report on the burden of KPn as part of a group with other Gram-negative bacteria. Nevertheless, *K. pneumoniae* has been reported as a common cause of presumed hospital-acquired infections (pHAI) and outbreaks in sub-Saharan Africa, including South Africa [8–12], and is often associated with resistance to multiple antibiotic classes. Investigation of the causes of childhood deaths using postmortem minimally invasive tissue sampling (MITs), implicated sepsis in the causal pathway to death in two-thirds of decedents, the major pathogens due to hospital-acquired organisms, including KPn [13, 14].

Despite the significant disease burden and rising antimicrobial resistance associated with *K. pneumoniae*, no licensed vaccines are currently available. Progress toward vaccine development has been hindered by the pathogen's extensive genetic diversity, complex virulence mechanism, and geographical variability in circulating strains [15]. Robust genomic surveillance—especially in Africa, where data remains limited and the burden of disease is disproportionately high—is needed for delineating the epidemiology of illness and identifying targets for potential interventions, including vaccine development.

This study aimed to genetically characterize KPn isolates associated with invasive disease, including fatal cases, over 6 years in South African infants.

## METHODS

### Study Design and Population

The analyzed KPn isolates were collected across 4 observational studies which included KPn surveillance. The first study was undertaken from March 4, 2019 to February 7, 2021 across 6 hospitals as described [16]. The second study was conducted from May 22, 2022 to December 31, 2023, and included surveillance at the same 6 sites as the earlier study. In both studies, detailed in the Supplementary text, infants hospitalized for presumed serious bacterial infection were evaluated at the discretion of the attending physician, which included blood culture and a lumbar puncture in young infants with suspected meningitis. Microbiological testing was done at the National Health Laboratory Service (NHLS), the sole laboratory servicing public health facilities in South Africa, which has standardized equipment across its laboratories. As standard of care, the recommended empiric antibiotic treatment for neonates admitted with suspected sepsis is ampicillin and gentamicin if hospitalized within 72 hours of birth or hospital admission. In some instances, repeat blood and CSF cultures were undertaken during hospitalization to evaluate the antibiotic response and/or investigate newly suspected sepsis events. Demographic and risk factors for invasive KPn disease were evaluated at admission, including data on the child's gender, mode of delivery, and gestational age at delivery.

We also analyzed KPn isolates from decedents in which the organism was attributed in the causal pathway of death. The decedents were enrolled between February 15, 2018 and April 18, 2023 from the Child Health and Mortality Prevention Study (CHAMPS) at the Soweto, South Africa site (www.champshealth.org). The CHAMPS study aims to determine the causes of under 5 childhood deaths and stillbirths across 7 LMICs, including South Africa. MITs is performed within 24 to 72 hours of death and samples including blood, CSF, and lung tissue are sent for culture. For each case, the cause of death (COD) is determined by a multidisciplinary panel constituted of local experts including neonatologists, pediatricians, microbiologists, and histopathologists. The determination of the cause of death (DeCoDe) panel evaluated all available antemortem and postmortem data and reported on the COD according to WHO recommended guidelines [17].

Additional KPn isolates from decedents not eligible for enrollment into the CHAMPS study, as they were from outside of the Health Demographic Surveillance Site used in the CHAMPS study, were also enrolled between April 17, 2018 and September 14, 2022. The decedents who were enrolled outside of the CHAMPS program had more targeted investigations focused on identifying infectious causes of death (ie, MITs-lite). The differences in the procedures used for the CHAMPS and MITs-lite surveillance are summarized in Supplementary Table 1. In both postmortem studies, clinical data of the decedents was obtained from clinical record review. A DeCoDe panel evaluated the MITs-lite cases to determine the causal pathway to death, as undertaken for the CHAMPS study.

### Patient Consent Statement

The Human Medical Human Research Ethics Committee of the University of Witwatersrand granted ethics consent for all studies, and relevant hospital management approvals were obtained before study initiation. Written informed consent was obtained from the mother/guardian.

### Study Procedures

To prepare whole-genome sequencing libraries, stored isolates were subcultured, and genomic DNA extracted using standard methods. NexteraXT libraries were prepared (Illumina, San Diego, CA) and sequenced on the MiSeq platform generating 2 X 300 base paired-end (PE) reads (Illumina, San Diego, CA). Following sequencing, the quality of the raw reads was assessed using FASTQC v0.12.1 and processed using the Jekesa pipeline. Trim Galore removed adapters, ambiguous reads, and low-quality bases (Q > 30 and length >50 bp). De novo assemblies were generated with SPAdes and Shovill, and QUAST 5 was used to evaluate their quality and to calculate assembly matrices. Sequences were uploaded onto the Pathogenwatch6 database for species confirmation and ConFindr was used to check for contamination. MLST and cgMLST predictions were performed using Klebsiella PasteurMLST and BIGSdb-Pasteur, respectively. Capsule K and O serotype predictions were made with Kaptive V3. Antimicrobial resistance genes identified with Kleborate compared with the curated version of the Comprehensive Antibiotic Resistance Database program including ResFinder 9. Virulence genes were identified by Kleborate using the BLASTn for Key loci. Phylogenetic analysis was conducted using wgMLST and cgMLST. The sARGs were investigated through ResFinder. iTOL 12 was used to generate and visualize the phylogenetic tree.

### Outcomes

The primary objective of the study was to genetically characterize KPn isolates associated with invasive disease, including fatal cases, over 6 years in South African children, stratified by pHAI and presumed community-associated infections (pCAI). As an exploratory analysis, we analyzed temporal changes in Kpn isolates collected over 6 years, focusing primarily on isolates collected at the Chris Hani Baragwanath Academic Hospital (CHBAH), where most isolates were accrued.

### Statistical Analysis

Multiple Kpn isolates collected from the same individual were sequenced only if collected more than 7 days apart, considering the high likelihood of the isolates being due to the same invasive disease episode. In cases where multiple isolates were sequenced from the same participant, only genetically distinct isolates were included. When KPn was cultured concurrently from different sites, sequencing was limited to a single isolate per participant, using a hierarchical approach of blood, CSF, and lung tissue. Early onset invasive disease was defined as culture-confirmed invasive KPn within 72 hours of birth, and late-onset disease was defined as episodes diagnosed from 72 hours until ≤90 days of age. Further, pCAI were defined as invasive KPn detected on admission or within 72 hours of hospitalization or if the death occurred in the community [18]. Whereas, pHAI was defined as an invasive KPn detected more than 72 hours after admission to the hospital or if the DeCoDe panel attributed nosocomial infection to the causal pathway of the death. Multidrug resistance (MDR) was defined as the presence of genetic markers in 3 or more antimicrobial classes. Clonotypes, defined as the composite of the sequence type (ST), K-locus, and O-locus for each strain, were reported to capture the interplay between lineage (ST) and key surface antigenic loci (K and O), which together more accurately reflect the heterogeneity and epidemiological features of the bacterial population than individual typing schemes alone.

Data was analyzed with Stata Version 11.0 (StataCorp, Texas, USA) and R Version 4.1.1 (Vienna, Austria). The findings from the 4 observational studies were aggregated in the main analysis. The distribution of the isolates stratified by each observational studies is shown in Supplementary Table 2. A multiple logistic regression model adjusting for possible covariates was used to compare the ST, K-loci, O-antigen types, antimicrobial resistance markers (ARM), and virulence factors between pHAI and pCAI isolates. Percentages were reported alongside adjusted odds ratios (aOR) and 95% confidence intervals (CI). *P*-values of 0.05 were considered significant and no adjustment was undertaken for multiplicity in this hypothesis-generating study.

## RESULTS

Overall, 630 invasive diseases Kpn isolates were identified during the 4 observational studies (402 identified during hospital surveillance and 228 from postmortem sampling); Table 1. Of those, 367 (58.3%) were sequenced. Reasons for sequencing not being undertaken included 84 isolates collected <7 days apart, 98 isolates not retrieved from the NHLS, and KPn not identified during subculture for 81 isolates. After sequencing, thirteen additional isolates were excluded as their genotypes were identical to an earlier isolate sequenced. Two decedents had a genomically identical KPn identified during antemortem and postmortem sampling, thus the postmortem isolates were excluded from further analysis. Lastly, 4.1% (15/367) of the isolates sequenced were identified as other Klebsiella species and excluded from further analysis. Two isolates from 4 different infants and 3 isolates from 1 infant, collected during hospital surveillance, were included in the final analysis as sequencing identified different genotypes. Demographic characteristics between isolates included in the final analysis (n = 337) and those not retrieved from the NHLS (n = 98) are detailed in Supplementary Table 3.

**Table 1.** Study Consort

	Total Number Of Isolates	Observation Study 1:	Observation Study 2:	Observation Study 3:	Observation Study 4:
		Hospital Surveillance	Hospital Surveillance	Postmortem Sampling—CHAMPS	Postmortem Sampling—MITs lite
		4 Mar 2019–27 Feb 2021	13 May 2022–31 Oct 2023	17 Jan 17–19 Ap 2023	21 Feb 2018–15 Sept 2022
*K. pneumonia* cultures	630	198	204	116^b^	112^b^
Collected <7 d apart	84	44	40	-	-
Case plate not retrieved from NHLS	98	43	11	22	22
*K. pneumonia* not grown during subculture	81	6	9	27	39
Cultures available for WGS	367	105	144	67	51
Same genome as first^a^	13	7	6	-	-
Other Klebsiella species	15	2	8	1	4
Same genome was collected during hospital surveillance	2	-	-	2	0
Cultures included in the final analysis	337	96	130	64	47

Abbreviations: CHAMPS, Child Health and Mortality Prevention Surveillance; MITs, Minimally invasive tissue sampling; NHLS, National Health Laboratory service.

^a^Interval of isolates (days): 8, 10, 12, 16, 21, 22, 23, 24, 32, 39.

^b^ *K. pneumoniae* isolates from decedents in which the organism was attributed to the causal pathway of death.

### Clinical and Demographic Characteristics

Of the 337 isolates included in the final analysis, 1.9% (7/337) were from stillbirths, 4.2% (14/337) from neonates <72 hours of age, 22.8% (77/337) from neonates 3 to ≤7 days of age, 44.8% (151/337) from neonates 7 to ≤28 days of age, and 26.4% (89/337) from infants 28 to ≤90 days of age. Fifty-seven percent (191/337) of the isolates were from males, while 28.8% (97/337) were collected from cases born to women living with HIV (ie, HIV-exposed; Supplementary Table 3). Overall, 70.0% (236/337), 11.6% (39/337), and 9.5% (32/337) of the sequenced isolates were cultured from blood, lung tissue, and CSF, respectively. Seventy-two percent (243/337) of the isolates were from infants born prematurely (<37 weeks gestation), while 82% (275/337) of the isolates were from pHAI episodes; Supplementary Table 4.

### Genomic Diversity and Phylogenetic Analysis

**Figure 1.**
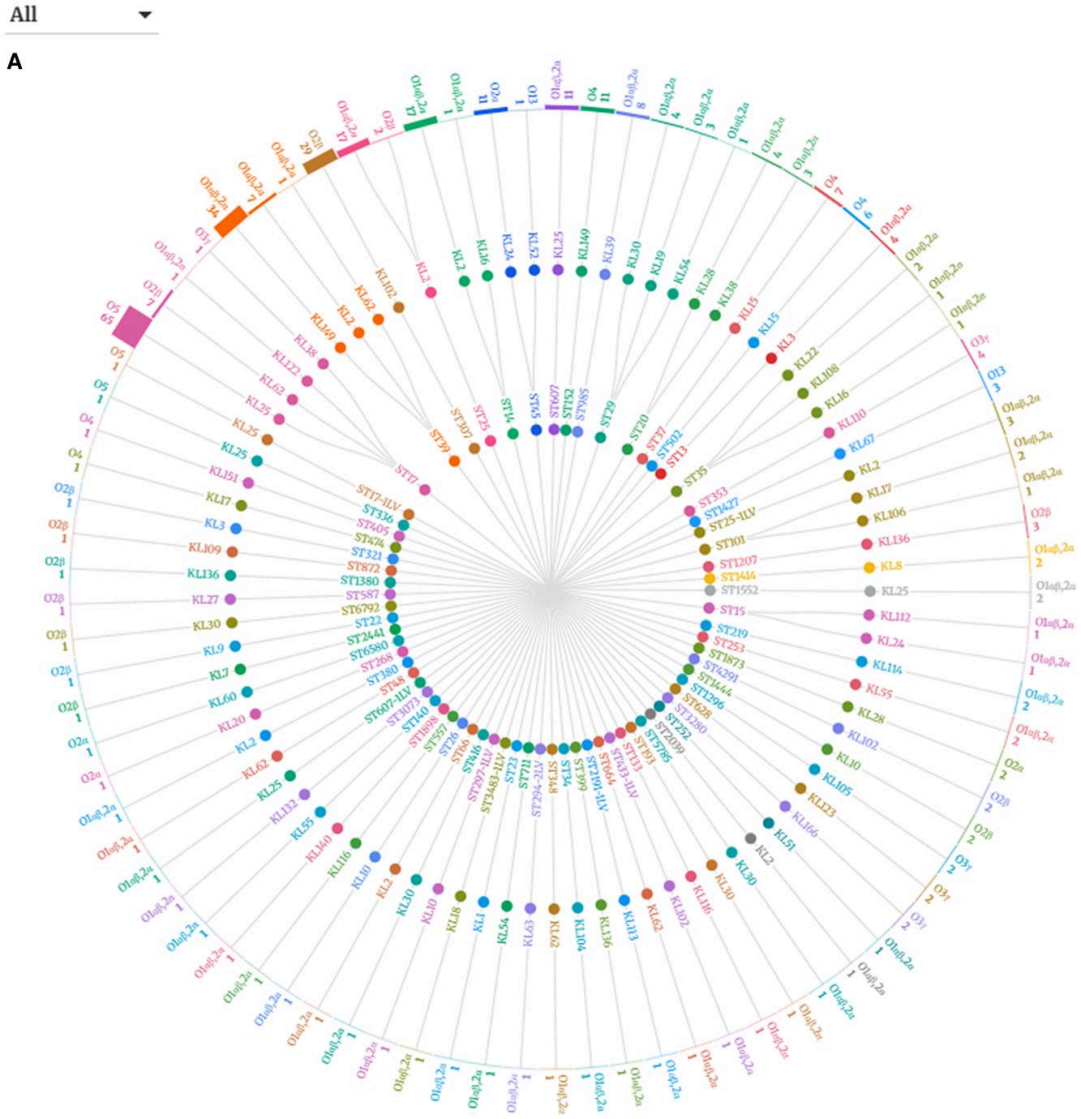

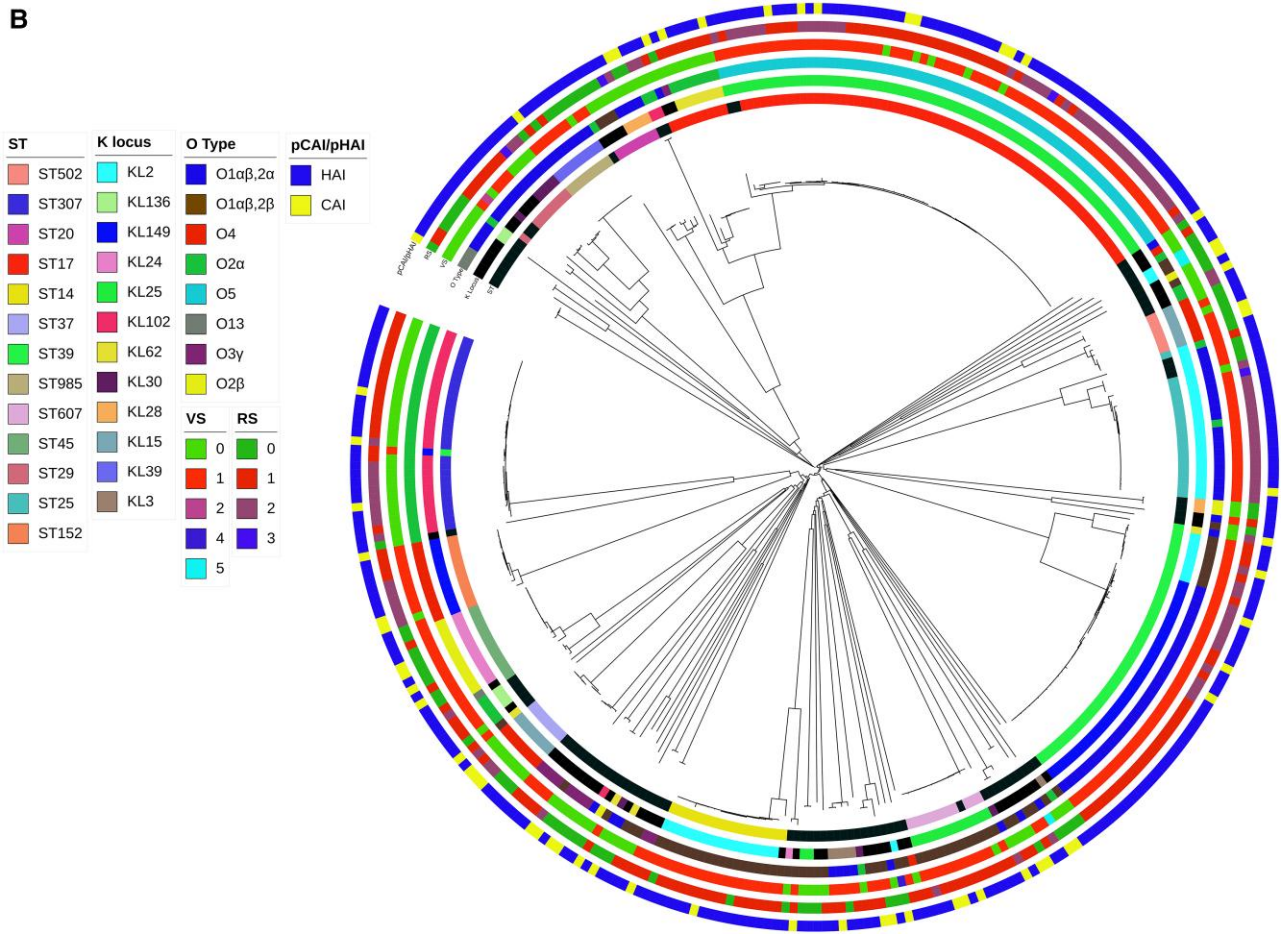
Hierarchical radical tree depicting the linkage between sequence types, K-loci, O-antigens (*A*), and Phylogenetic tree of *K. pneumoniae* (*B*).

There was a large diversity among KPn clones detected, with the majority (90.6%, 77/85) of clonotypes each contributing individually to <3% of the overall isolates; Figure 1*A*. In total, 48 K-loci and 7 O-antigens, with 85 combinations (ie, clonotypes), were identified among the 70 ST genomes. The most prevalent clonotype identified was ST17 associated with K-locus (KL) 25 and the O5 (19.3%, 65/337) antigen, followed by ST39 with KL149 and O1αβ,2α (9%, 34/377). Phylogenetic analysis showed historical branching and descent from common ancestral strains (Figure 1*B*). There are 3 major clades in the tree, with the major clade comprising isolates associated with fatal outcomes, suggesting that these isolates might be genetically closer (ie, have a common lineage) compared with those forming the other clusters. The 3 major clades were clustered based on the O-antigen and K-locus with O5, O1αβ,2α, and O2β clustering with KL25, KL149, and KL102 respectively.

**Figure 2.**
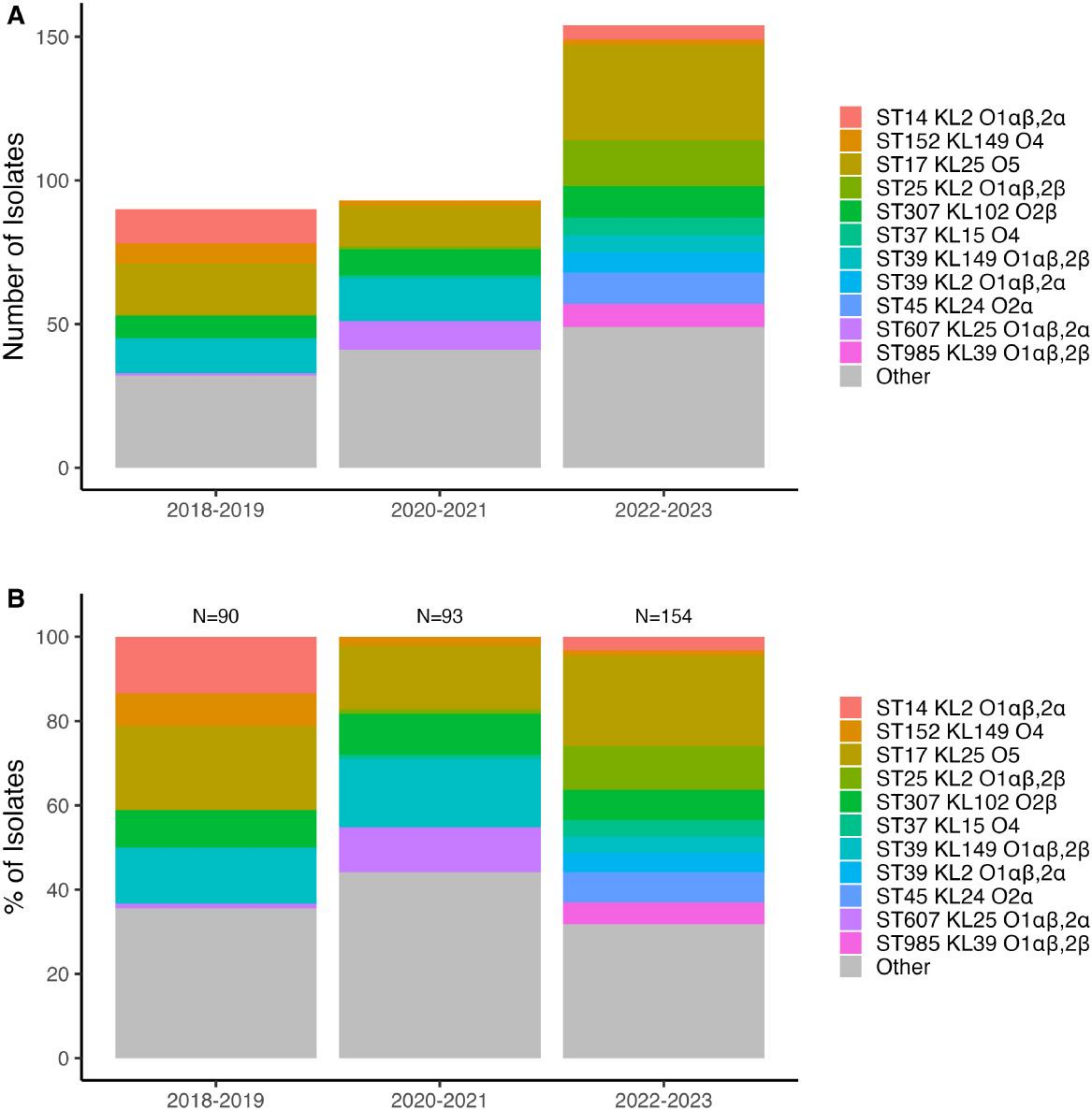
Changes in *K. pneumoniae* clonotypes causing invasive disease in South African infants.

The distribution of the Kpn strains causing invasive disease collected from CHBAH varied across the 6 years (Figure 2). While ST17 isolates harboring the KL25 capsule and O5 antigen were detected throughout the study period, ST39-KL149-O1αβ,2α (80.8%, 21/26) and ST307-KL102-O2β (52.0%, 13/25) clonotypes were mainly detected between October 2019 and June 2020, respectively. The distribution of clonotypes, ST, K-loci, and O-antigens at the different collection sites is detailed in the Supplementary text and Supplementary Figure 1.

### ST, Capsular Polysaccharide (K-) Loci, and Lipopolysaccharide (O-) Antigens

Overall, ST17 predominated (22.0%, 74/337), followed by ST39 (12.5%, 42/337; Table 2). There were no statistical differences in the prevalence of the STs between pHAI and pCAI isolates; however, there was a greater diversity of STs in pHAI (n = 46) than in pCAI (n = 21) isolates; Supplementary Figure 2a. Overall, KL25 predominated (24.0%, 81/337), followed by KL2 (14.5%, 49/337), KL149 (13.4%, 45/337), and KL102 (9.5%, 32/337; Table 2). The dominant O-antigen detected was O1αβ,2α (48.1%, 162/337) followed by O5 (19.9%, 67/337; Table 2). The prevalence of the K-loci and O-antigens was similar between pCAI and pHAI isolates, except for a lower prevalence of KL62 (2.2%, 6/275 vs 8.1%, 5/62; *P* = .034) and O3γ in pHAI isolates (2.2%, 6/275 vs 8.1%, 5/62; *P* = .027), respectively; Supplementary Figure 2b and c.

**Table 2.** Genomic Characterization of *K. pneumoniae* Causing Invasive Disease and Mortality in South African Infants 0–90 Days of Age

	Overall (N = 337)	PCAI (N = 62)	PHAI (N = 275)	OR; 95% CI; *P*-value	AdjOR; 95% CI; *P*-value
Sequence types (ST), n (%)					
ST14	18 (5.34)	1 (1.61)	17 (6.18)	4.01 (.60–170.58); .214	9.26 (1.1–356.19); .113
ST152	11 (3.26)	3 (4.84)	8 (2.91)	0.59 (.14–3.56); .432	1.25 (.25–11.18); .812
ST17	74 (21.96)	11 (17.74)	63 (22.91)	1.38 (.66–3.11); .497	1.49 (.7–3.5); .324
ST20	7 (2.08)	2 (3.23)	5 (1.82)	0.56 (.09–5.98); .617	–
ST25	19 (5.64)	1 (1.61)	18 (6.55)	4.26 (.65–180.76); .218	17.64 (.99–2245.18); .171
ST29	8 (2.37)	0 (0)	8 (2.91)	–	–
ST307	29 (8.61)	3 (4.84)	26 (9.45)	2.05 (.60–10.93); .32	3.37 (.76–30.71); .181
ST37	7 (2.08)	3 (4.84)	4 (1.45)	0.29 (.05–2.04); .119	.26 (.05–1.93); .136
ST39	42 (12.46)	5 (8.06)	37 (13.45)	1.77 (.65–6.03); .293	–
ST45	12 (3.56)	4 (6.45)	8 (2.91)	0.44 (.11–2.04); .244	–
ST607	11 (3.26)	3 (4.84)	8 (2.91)	0.59 (.14–3.56); .432	1.01 (.25–5.48); .989
ST985	8 (2.37)	1 (1.61)	7 (2.55)	1.59 (.20–72.95); >.999	–
Other ST^a^	91 (27)	25 (40.32)	66 (24)	0.47 (.25–.88); .011	0.43 (.23–.82); .009
K-loci, n (%)					
KL102	32 (9.5)	3 (4.84)	29 (10.55)	2.31 (.68–12.27); .231	3.75 (.85–34.24); .146
KL149	45 (13.35)	6 (9.68)	39 (14.18)	1.54 (.61–4.67); .414	1.65 (.63–5.36); .349
KL15	13 (3.86)	5 (8.06)	8 (2.91)	0.34 (.09–1.38); .07	0.34 (.1–1.33); .097
KL2	49 (14.54)	5 (8.06)	44 (16)	2.17 (.81–7.32); .161	2.31 (.81–8.89); .161
KL24	12 (3.56)	4 (6.45)	8 (2.91)	0.44 (.11–2.04); .244	–
KL25	81 (24.04)	11 (17.74)	70 (25.45)	1.58 (.76–3.56); .25	1.86 (.88–4.34); .124
KL30	8 (2.37)	1 (1.61)	7 (2.55)	1.59 (.20–72.95); >.999	4.62 (.48–191.09); .293
KL39	8 (2.37)	1 (1.61)	7 (2.55)	1.59 (.20–72.95); >.999	–
KL62	11 (3.26)	5 (8.06)	6 (2.18)	0.26 (.06–1.10); .034	–
Other K-loci^a^	78 (23.15)	21 (33.87)	57 (20.73)	0.51 (.27–.99); .031	0.52 (.27–1.01); .05
O-type, *n* (%)					
O13	4 (1.19)	1 (1.61)	3 (1.09)	0.67 (.05–35.89); .558	–
O1αβ,2α	162 (48.07)	29 (46.77)	133 (48.36)	1.07 (.59–1.93); .888	1.1 (.6–2.04); .761
O2α	15 (4.45)	5 (8.06)	10 (3.64)	0.43 (.13–1.67); .165	–
O2β	52 (15.43)	6 (9.68)	46 (16.73)	1.87 (.75–5.63); .241	1.92 (.74–6.2); .222
O3γ	11 (3.26)	5 (8.06)	6 (2.18)	0.26 (.06–1.10); .034	0.22 (.06–.91); .027
O4	26 (7.72)	8 (12.9)	18 (6.55)	0.47 (.18–1.33); .111	0.65 (.25–1.97); .416
O5	67 (19.88)	8 (12.9)	59 (21.45)	1.84 (.81–4.73); .159	1.9 (.83–4.99); .154
Multidrug resistance (MDR), n (%)	287 (85.16)	47 (75.81)	240 (87.27)	2.18 (1.02–4.50); .029	2.69 (1.27–5.52); .008
Aminoglycosides	285 (84.57)	46 (74.19)	239 (86.91)	2.30 (1.10–4.68); .018	2.78 (1.34–5.65); .005
Carbapenems	112 (33.23)	17 (27.42)	95 (34.55)	1.40 (.74–2.75); .301	2.06 (1.01–4.54); .058
Third-generation cephalosporins	256 (75.96)	40 (64.52)	216 (78.55)	2.01 (1.05–3.78); .031	2.81 (1.46–5.38); .002
Colistin	8 (2.37)	1 (1.61)	7 (2.55)	1.59 (.20–72.95); >.999	–
Fluoroquinolones	166 (49.26)	26 (41.94)	140 (50.91)	1.43 (.79–2.62); .209	2 (1.06–3.92); .036
Fosfomycin	0 (0)	0 (0)	0 (0)	–	–
Penicillins	327 (97.03)	60 (96.77)	267 (97.09)	1.11 (.11–5.77); >.999	–
Penicillin (beta-lactamase inhibitors)	2 (0.59)	1 (1.61)	1 (.36)	0.22 (.00–17.76); .335	–
Amphenicols	202 (59.94)	29 (46.77)	173 (62.91)	1.93 (1.07–3.50); .022	2.35 (1.25–4.49); .008
Sulfonamides	251 (74.48)	44 (70.97)	207 (75.27)	1.24 (.63–2.37); .52	1.7 (.86–3.28); .116
Tetracycline	70 (20.77)	14 (22.58)	56 (20.36)	0.88 (.44–1.85); .73	.87 (.42–1.93); .72
Tigecycline	0 (0)	0 (0)	0 (0)	–	–
Trimethoprim	251 (74.48)	45 (72.58)	206 (74.91)	1.13 (.57–2.17); .748	1.49 (.75–2.87); .244
Virulence factors, n (%)					
ybt	218 (64.69)	36 (58.06)	182 (66.18)	1.41 (.77–2.57); .241	1.33 (.71–2.48); .369
clb	4 (1.19)	2 (3.23)	2 (.73)	0.22 (.02–3.11); .156	–
iuc	4 (1.19)	3 (4.84)	1 (0.36)	0.07 (.00–.92); .021	–
Virulence factor combinations, n (%)					
ybt only	214 (63.5)	33 (53.23)	181 (65.82)	1.69 (.93–3.06); 0.079	1.65 (.88–3.06); .113
ybt and clb	4 (1.19)	2 (3.23)	2 (0.73)	0.22 (.02–3.11); .156	–
iuc only	0 (0)	0 (0)	0 (0)	–	–
icu and ybt w/o clb	1 (0.3)	1 (1.61)	0 (0)	0.00 (.00–8.79); .184	–
ybt, clb, and iuc	3 (0.89)	2 (3.23)	1 (0.36)	0.11 (.00–2.16); .088	–
AMR and virulence factor combinations, n (%)					
Carbapenems and ybt	78 (23.15)	7 (11.29)	71 (25.82)	2.73 (1.17–7.43); .013	4.18 (1.59–14.49); .009
Carbapenems and clb	0 (0)	0 (0)	0 (0)	–	–
ESBL and ybt	185 (54.9)	26 (41.94)	159 (57.82)	1.89 (1.05–3.46); .025	2.21 (1.19–4.2); .013
ESBL and clb	1 (0.3)	0 (0)	1 (0.36)	–	–

Abbreviations: adjOR, adjusted odd ratio; clb, colibactin; ESBL, extended spectrum β-lactamase; iuc, aerobactin; OR, odd ratio; ST, sequence type; ybt, yersiniabactin.

Presumed hospital-acquired infections (pHAI) were defined as an invasive *K. pneumoniae* detected more than 72 hours after admission to the hospital or if the DeCoDe panel attributed nosocomial infection to the causal pathway of the death.

Presumed community-acquired infections (pCAI) were defined as invasive *K. pneumoniae* detected within 72 hours of hospitalization or if the death occurred in the community.

^a^Sequence types and K-loci contributing <2% of the total 337 isolates.

Other STs: ST101; ST1207; ST1296; ST13; ST133; ST1380; ST140; ST1414; ST1427; ST1444; ST15; ST1552; ST17-1LV; ST1873; ST1898; ST193; ST2039; ST219; ST2191-1LV; ST22; ST23; ST2441; ST25-1LV; ST252; ST253; ST26; ST268; ST294-2LV; ST297-1LV; ST3073; ST321; ST3280; ST336; ST34; ST348; ST3483-1LV; ST35; ST353; ST380; ST399; ST405; ST416; ST4291; ST433-1LV; ST474; ST48; ST502; ST557; ST5785; ST587; ST607-1LV; ST628; ST6580; ST66; ST664; ST6792; ST711; ST872.

Other K-loci: KL1; KL10; KL104; KL105; KL106; KL108; KL109; KL110; KL112; KL113; KL114; KL116; KL122; KL123; KL132; KL136; KL140; KL151; KL16; KL166; KL17; KL18; KL19; KL20; KL22; KL27; KL28; KL3; KL38; KL51; KL52; KL54; KL55; KL60; KL63; KL67; KL7; KL8; KL9; Unknown.

Adjusted odd ratio and 95% CI calculated using logistic regression analyses. *P*-values of <.05 are considered significant.

–, too few variables to calculate.

The prevalence of ST, K-loci, and O-antigens stratified by collection site is detailed in Supplementary Table 5.

### Antimicrobial Resistance

Overall, 85.2% (287/337) of the KPn isolates harbored MDR genes, including 84.6% (285/337) harboring genes encoding resistance to aminoglycosides, 76.0% (256/337) to 3rd generation cephalosporins, 33.2% (112/337) to carbapenems, and 2.4% (8/337) to colistin (Table 2). There was a 2.69 higher odds of MDR genes in pHAI (87.3%, 240/275) compared with pCAI (75.8%, 47/62; *P* = .008) isolates, including a higher prevalence of genes conferring resistance to aminoglycosides (86.9%, 239/275 vs 74.1%, 46/62; *P* = .005) and 3rd generation cephalosporins (78.6%, 216/275 vs 64.5%, 40/62; *P* = .002; Table 2).

Overall, the TEM-1D (bla_TEM-1D)_ and CTX-M-15 (bla_CTX-M-15_) β-lactamase genes were detected in 61.4% (207/337) and 75.1% (253/337) of the isolates, respectively whilst the oxacillinase-type β-lactamase-48 (bla_OXA-181_) gene was detected in 26.4% (89/337) of the isolates. The New Delhi metallo β-lactamase-5 (bla_NDM-5_) and New Delhi metallo β-lactamase-1 (bla_NDM-1_) genes were detected in 2.1% (7/337) and 0.3% (1/337) of the isolates, respectively (Supplementary Table 6). The prevalence of the antimicrobial resistance genes was similar between pHAI and pCAI isolates except for a higher prevalence of bla_TEM-1D_ (64.4%, 177/275 vs 48.4%, 30/62; *P* = .021) and bla_CTX-M-15_ (77.8%, 214/275 vs 62.9%, 39/62; *P* = .022) in pHAI compared with pCAI, respectively.

**Figure 3.**
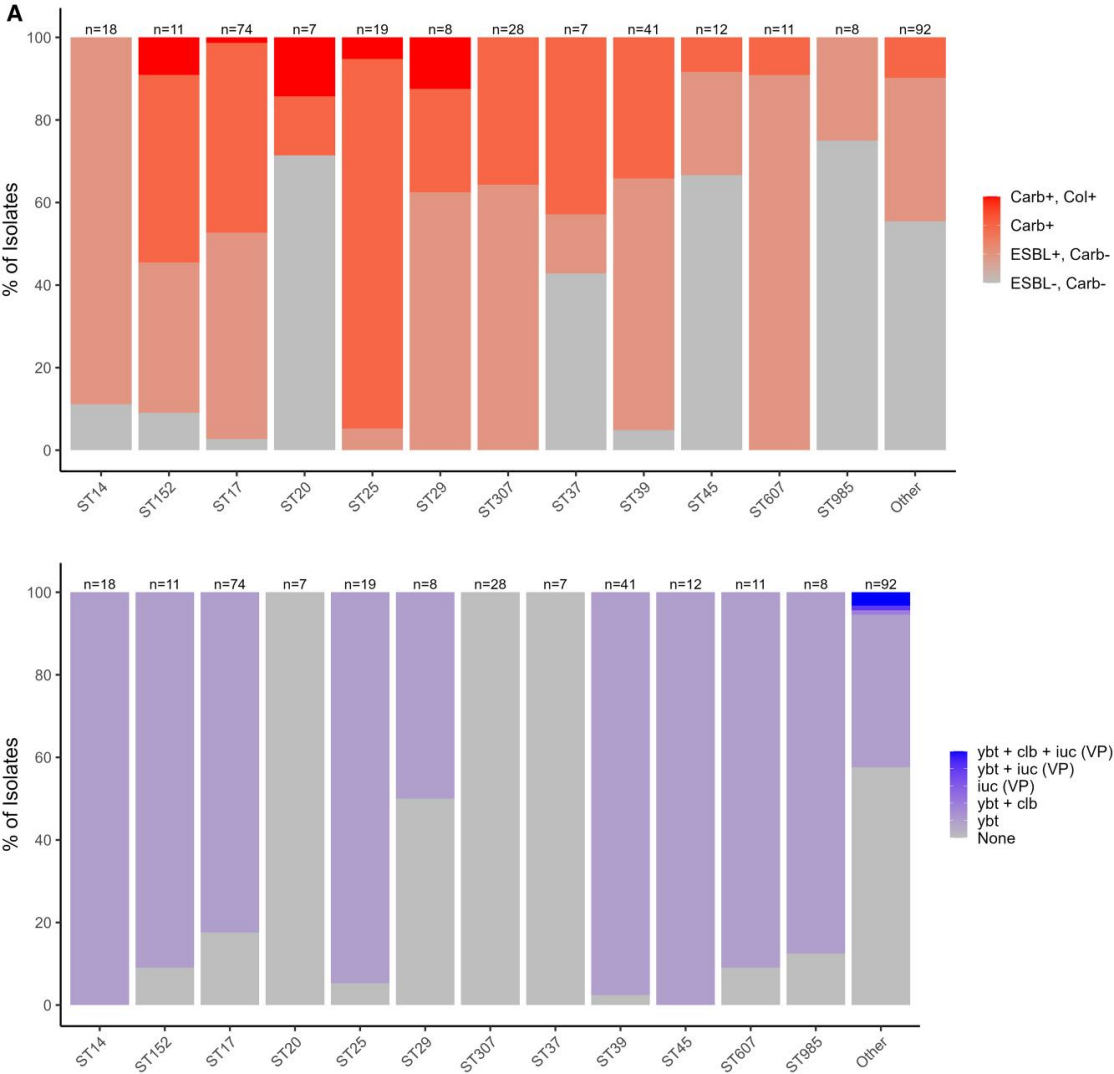

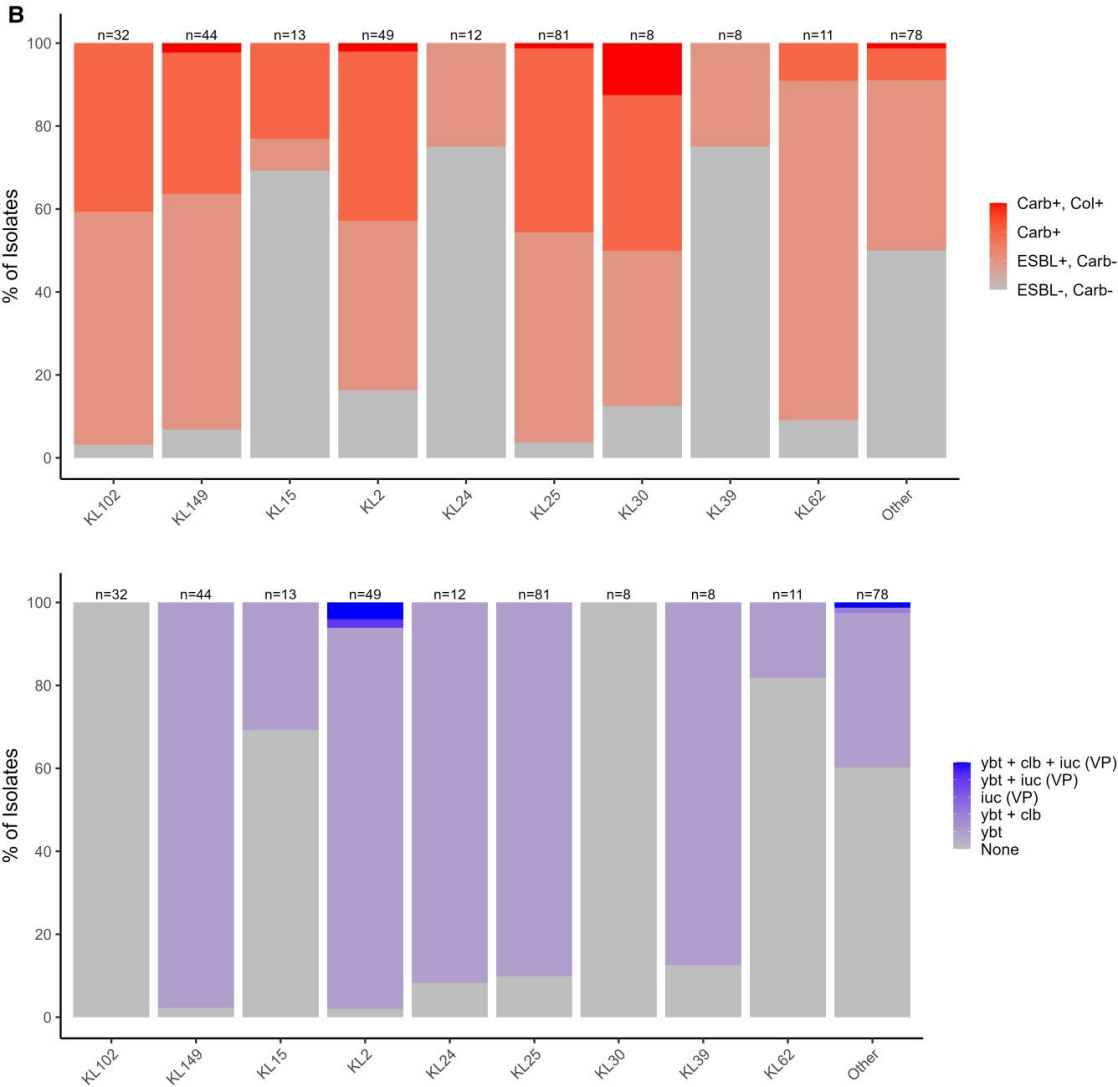

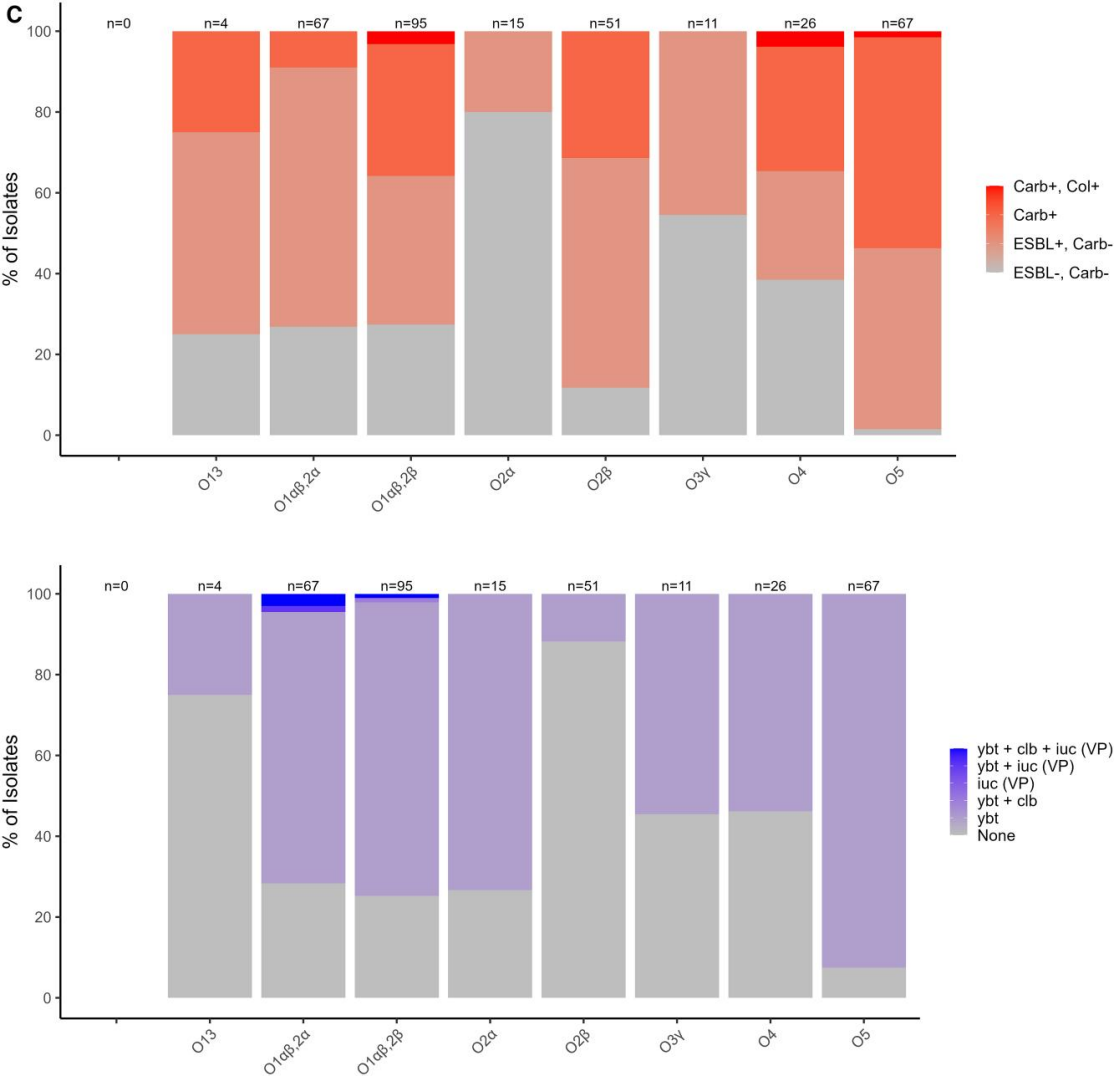

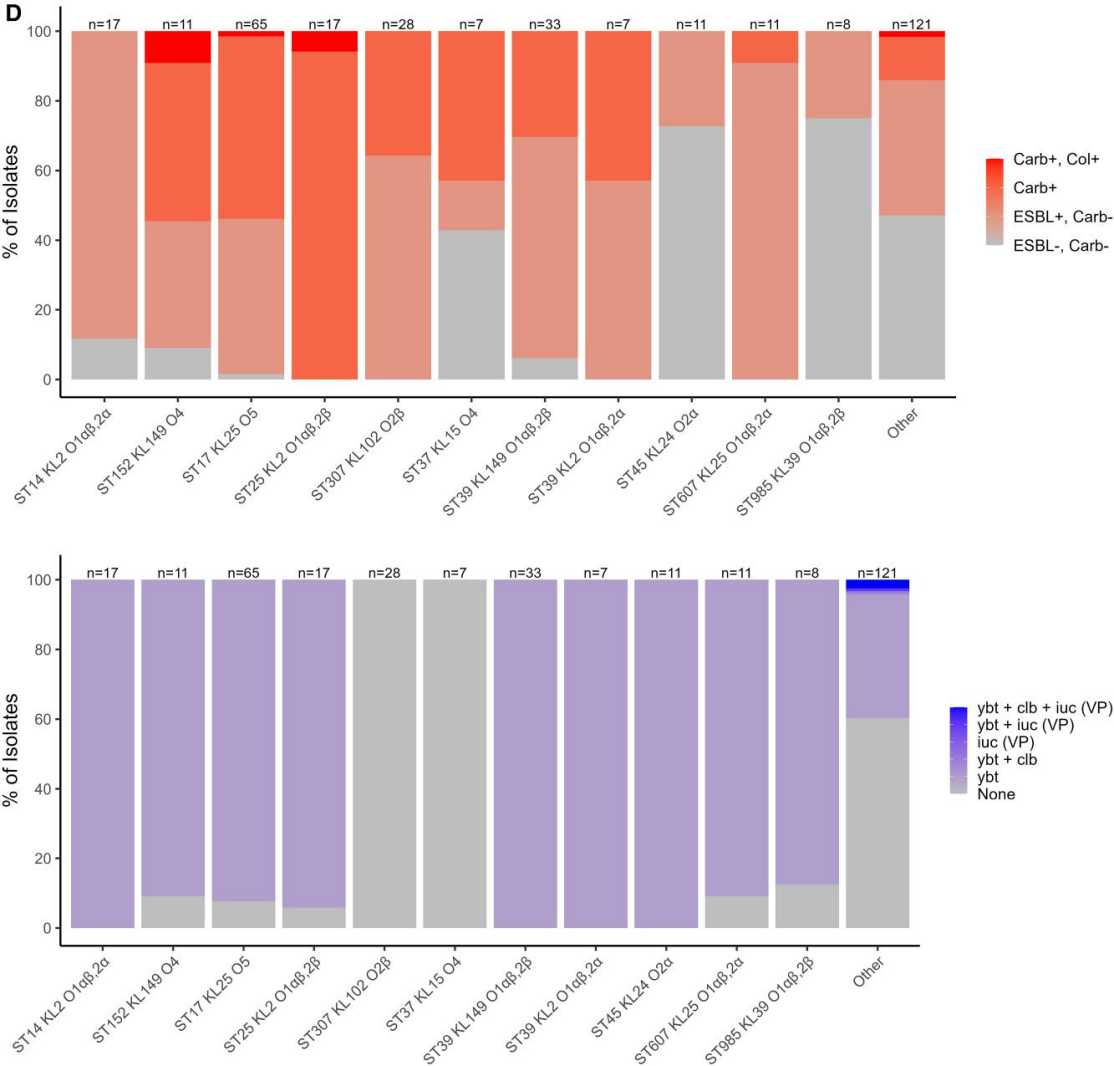
Distribution of resistance and virulence scores, stratified by common ST (*A*), K-loci (*B*), and O-antigens (*C*) and clonotypes (*D*).

The resistance scores, which identify clonotypes warranting escalation of antimicrobial therapy [19], varied by clonotype, ST, K-loci, and O-loci (Figure 3*A*-*D*). The prevalence of genes encoding resistance to Extended-Spectrum Beta-Lactamases (ESBL) production was highest in ST607 (90.9%, 10/11), KL62 (81.8%, 9/11), and O2β (57.7%, 30/52) and lowest in ST20 (0%, 0/7) and ST502 (0%, 0/6), KL15 (7.7%, 1/13), and O2αγ (15%, 3/15) isolates, respectively (Figure 3*A*–*C*). The prevalence of genes encoding resistance to carbapenem was highest in ST1444 (100%, 2/2), ST25 (94.7%, 18/19), KL25 (51.8%, 42/81), and O5 (61.2%, 41/67), respectively. A single ST25 isolate, as well as 2 KL25 (2.5%; 2/81), 2 KL2 (4.1%; 2/49), and a single KL30 (12.5%; 1/8) isolate, respectively harbored genes encoding resistance to both carbapenem and colistin.

When stratifying by the most identified clonotypes, all ST25-KL2-O1αβ,2α isolates (n = 17) harbored genes associated with carbapenem resistance, including a single isolate that also harbored genes for colistin resistance; Figure 3*D*. Other differences are detailed in the supplementary text and Supplementary Figure 3.

### Virulence Factors

Yersiniabactin (ybt), associated with hypervirulence, was the dominant virulence factor detected in 64.7% (218/337) of the isolates, while genes encoding for colibactin (clb, 4/337) and aerobactin (iuc, 4/337) virulence were detected in <2% of isolates; Table 2. Twenty-three percent (78/337) of the isolates harbored genes encoding for ybt virulence and carbapenems resistance. There was a 4.18 higher odds of harboring genes encoding for both ybt virulence and carbapenems resistance in pHAI (25.8%, 71/275) compared with pCAI (11.3%, 7/62; *P* = .009) isolates (Table 2). Fifty-five percent (185/337) of isolates harbored genes encoding for both ybt and ESBL production, with a higher prevalence in pHAI (57.8%, 159/275) compared with pCAI (41.9%, 26/62; aOR 2.21; 95% CI:1.19–4.2 *P* = .013) isolates. The distribution of virulence factors stratified by clonotypes, ST, K-loci, and O-antigens is illustrated in Figure 3*A*-*D*

## DISCUSSION

We documented a wide diversity of MDR KPn strains associated with invasive disease in South African infants up to 90 days of age between 2018 and 2023. We also identified several high-risk clonotypes, including ST25-KL2-O1αβ,2α, with all isolates harboring genes associated with carbapenem resistance. Furthermore, the ST307-KL102-O2β, ST39-KL149-O1αβ,2α, ST607-KL25-O1αβ,2α, ST17-KL25-O5, and ST152-KL149-O4 clonotypes were associated with a high prevalence of genes encoding for ESBL production or carbapenem resistance. Most ST25, ST607, ST307, ST39, ST152, and ST17 clones also harbored genes for ybt (81%–100%) virulence. While the ST25, ST17, ST307, and ST39 clones have been reported elsewhere, including South Africa [20–22], the ST607 clones are rare and have only been reported in a limited number of invasive isolates from China and France [23].

Overall, most of the KPn isolates sequenced harbored MDR genes, spanning resistance to first, second, and third-line therapeutic options. This aligns with global reports of increased acquisition of antibiotic resistance and the broader antimicrobial resistance landscape in South Africa and poses significant challenges for managing KPn infections in our setting [24, 25]. The emergence of MDR and highly virulent strains harboring genes for ybt virulence along with ESBL production or carbapenems resistance is particularly worrying in terms of the clinical ramifications of increased severity of infections, limited treatment options, increased case-fatality risk, and potential for rapid dissemination within healthcare facilities [26, 27]. In South Africa, the standard treatment for young infants suspected of having community-acquired sepsis includes ampicillin and gentamicin treatment while pHAI is treated with third-generation cephalosporins or carbapenems. The AMR profile of the sequenced Kpn strains in this study indicates that these treatment options could largely be ineffective, underscoring the critical need for vaccines or alternative, more effective drug combinations.

Although the prevalence of ST, K-loci, and O-antigens was similar between pHAI and pCAI isolates, pHAI isolates had a 2.69 higher odd of harboring MDR genes and a 4.18 higher odd of harboring genes encoding for both ybt virulence and carbapenems resistance compared with pCAI isolates. The heightened prevalence of MDR among isolates acquired within healthcare settings emphasizes the significant role that healthcare facilities play in the spread of antimicrobial resistance. It is, however, noteworthy that 76% of the pCAI isolates sequenced also harbored MDR genes, which may be attributed to high exposure to antibiotics in our setting or dissemination of pHAI strains in the community.

Studies are underway on developing a vaccine to protect against the most common Kpn strains; however, the geographic variability in strain distribution poses a significant challenge to vaccine development [15]. In our setting, a vaccine achieving over 80% coverage would need to target 11 capsular serotypes to protect against invasive disease. The dominant capsular serotypes identified in our setting included KL25, KL149, KL2, KL102, and KL15, which largely align with global distributions [28], except for KL149, reported only in a few studies from Africa [29–31] and Poland [32]. The major K-loci detected in our setting and globally are important targets for consideration for multivalent K-antigen vaccine development. Genomic characterization of Kpn invasive disease in adults across Africa remains limited often restricted to small datasets or selectively sequenced AMR strains. Nevertheless, a recent study in Ghana identified KL116, KL2, and KL102 as predominant capsular types in a mixed pediatric and adult cohort [33]. Among hospitalized adults in South Africa, KL64, KL102, and KL25 were the most prevalent carbapenemase-producing K-loci strains in Gauteng [34], whereas KL114, KL102, and KL25 predominated in Cape Town [20]. These findings suggest that vaccine targets identified in pediatric populations may also be relevant for protecting adults in South Africa; however, given the relatively small sample sizes of available South African studies (n = 24 and n = 85), further large-scale genomic investigations are needed to understand the diversity and distribution of *K. pneumoniae* capsular types and to confirm the potential overlap in vaccine targets between pediatric and adult populations. Moreover, variations in KPn lineages observed in this study and over 20 years in Malawi [35] underscore the need for ongoing surveillance if a multivalent K-antigen-based vaccine is developed, considering the fluctuation of dominant lineages over time and the potential for serotype replacement disease by nonvaccine serotypes. A vaccine targeting the lipopolysaccharide serotypes, which are fewer in number and remain more stable throughout the study period, may be less prone to replacement disease by nonvaccine serotypes, with the inclusion of 3 O-antigens (O1αβ,2α, O2β, and O5) covering over 80% of strains in our study.

Limited studies have characterized colonizing KPN isolates in Africa, including South Africa, primarily due to challenges in surveillance and diagnostic infrastructure. Nevertheless, a previous study by our group examining the genomic relatedness between a limited number of colonizing and invasive Kpn strains in South African infants indicated that there might be some genomic differences between these groups [36]. The observations included that invasive strains compared with colonizing strains had a narrower range of lineages, higher prevalence of multidrug resistance (MDR) genes, and higher prevalence of ybt; however, further research is needed to fully elucidate the relationship between colonizing and invasive strains, particularly in African settings.

Strengths of this study included that isolates of KPn were identified through both observational surveillance and postmortem tissue sampling, and were not biased toward selecting isolates associated with MDR. Moreover, isolates were collected from young infants (<90 days of age), with most other studies focusing on older children and adults. Lastly, a large number of isolates were sequenced with appropriate associated clinical and demographic data.

Limitations of this study included that most of the KPn strains sequenced were identified at a single site (CHBAH), limiting the generalizability of the data to a national level. Further, not all *K. pneumoniae* case plates could be retrieved from the reference laboratory for downstream sequencing. Consequently, our results may not fully capture the complete genomic diversity of *K. pneumoniae* disease, and findings should therefore be interpreted with this consideration in mind. There were limited differences in the clinical characteristics between cases with and without available isolates, suggesting that any resulting bias is likely to be limited. There is also some uncertainty in the definitions used to classify infections, as pCAI and pHAI, which may not conclusively indicate the source of infection. The sample size for pCAI isolates was small, thus results should be interpreted with caution. Also, phenotypic susceptibility results were unavailable and could not be cross-checked against the genotypic ARM data. This study was hypothesis generating and not powered to investigate individual differences in ST, K-loci, and O-antigen, and we thus did not adjust for multiplicity. Lastly, antemortem sampling alone has low sensitivity compared with combined antemortem and postmortem sampling, where multiple sites are sampled and not restricted by low blood volume. Consequently, data from the 4 observational studies were aggregated for the main analysis; however, this may have introduced some bias weighted toward more virulent Kpn strains or those not covered by standard empirical antimicrobial regimens and findings should be interpreted with caution.

In conclusion, although there was a wide diversity of strains associated with Kpn invasive disease in South African children, over 80% of the isolates were attributed to 11 capsular loci or 3 lipopolysaccharide (O-antigen) serotypes. The findings from this study could be useful in selecting KPn antigen targets for potential vaccine candidates. Moreover, the changing epidemiology of MDR KPn causing invasive disease in South African infants emphasizes the need for continuous genomic characterization in LMIC regions to track changes in virulence and antibiotic susceptibility profiles.

## Supplementary Material

ofag004_Supplementary_Data

## Supplementary Data

Supplementary materials are available at *Open Forum Infectious Diseases* online. Consisting of data provided by the authors to benefit the reader, the posted materials are not copyedited and are the sole responsibility of the authors, so questions or comments should be addressed to the corresponding author.

## References

[1] Liu L, Oza S, Hogan D, et al Global, regional, and national causes of under-5 mortality in 2000–15: an updated systematic analysis with implications for the Sustainable Development Goals. Lancet 2016; 388:3027–35. 27839855 10.1016/S0140-6736(16)31593-8 PMC5161777

[2] Liang L, Kotadia N, English L, et al Predictors of mortality in neonates and infants hospitalized with sepsis or serious infections in developing countries: a systematic review. Frontiers Pediatr 2018; 6:277. 10.3389/fped.2018.00277 PMC6190846 30356806

[3] Wang H, Abajobir AA, Abate KH, et al GBD 2016 mortality collaborators global, regional, and national under-5 mortality, adult mortality, age-specific mortality, and life expectancy, 1970–2016: a systematic analysis for the Global Burden of Disease Study 2016. Lancet 2017; 390:1084–150. 28919115 10.1016/S0140-6736(17)31833-0 PMC5605514

[4] Waters D, Jawad I, Ahmad A, et al Aetiology of community-acquired neonatal sepsis in low and middle income countries. J Glob Health 2011; 1:154. 23198116 PMC3484773

[5] Cohen-Wolkowiez M, Moran C, Benjamin DK, et al Early and late onset sepsis in late preterm infants. Pediatr Infect Dis J 2009; 28:1052–6. 19953725 10.1097/inf.0b013e3181acf6bd PMC2798577

[6] Stoll BJ, Hansen NI, Sánchez PJ, et al Early onset neonatal sepsis: the burden of group B streptococcal and E. coli disease continues. Pediatrics 2011; 127:817–26. 21518717 10.1542/peds.2010-2217 PMC3081183

[7] UNICEF and World Health Organization . Ending preventable newborn and stillbirths by 2030: moving faster towards high-quality universal health coverage in 2020–2025. USA: UNICEF and World Health Organization, 2020.

[8] Gorrie CL, Mirčeta M, Wick RR, et al Genomic dissection of Klebsiella pneumoniae infections in hospital patients reveals insights into an opportunistic pathogen. Nat Commun 2022; 13:1–17. 35641522 10.1038/s41467-022-30717-6 PMC9156735

[9] Gezmu AM, Bulabula ANH, Dramowski A, et al Laboratory-confirmed bloodstream infections in two large neonatal units in sub-Saharan Africa. Int J Infect Dis 2021; 103:201–7. 33227511 10.1016/j.ijid.2020.11.169

[10] Reddy K, Bekker A, Whitelaw AC, Esterhuizen TM, Dramowski A. A retrospective analysis of pathogen profile, antimicrobial resistance and mortality in neonatal hospital-acquired bloodstream infections from 2009–2018 at Tygerberg Hospital, South Africa. PLoS One 2021; 16:e0245089. 33444334 10.1371/journal.pone.0245089 PMC7808607

[11] Essel V, Tshabalala K, Ntshoe G, et al A multisectoral investigation of a neonatal unit outbreak of Klebsiella pneumoniae bacteraemia at a regional hospital in Gauteng Province, South Africa. S Afr Med J 2020; 110:783–90. 32880307 10.7196/SAMJ.2020.v110i8.14471

[12] Chung The H, Karkey A, Pham Thanh D, et al A high-resolution genomic analysis of multidrug-resistant hospital outbreaks of Klebsiella pneumoniae. EMBO Mol Med 2015; 7:227–39. 25712531 10.15252/emmm.201404767 PMC4364942

[13] Madhi SA, Pathirana J, Baillie V, et al Unraveling specific causes of neonatal mortality using minimally invasive tissue sampling: an observational study. Clin Infect Dis 2019; 69:S351–s360. 31598660 10.1093/cid/ciz574 PMC6785687

[14] Taylor AW, Blau DM, Bassat Q, et al Initial findings from a novel population-based child mortality surveillance approach: a descriptive study. Lancet Glob Health 2020; 8:e909–19. 32562647 10.1016/S2214-109X(20)30205-9 PMC7303945

[15] Dangor Z, Benson N, Berkley JA, et al Vaccine value profile for Klebsiella pneumoniae. Vaccine 2024; 42:S125–41. 38503661 10.1016/j.vaccine.2024.02.072

[16] Madhi SA, Anderson AS, Absalon J, et al Potential for maternally administered vaccine for infant Group B Streptococcus. N Engl J Med 2023; 389:215–27. 37467497 10.1056/NEJMoa2116045

[17] Verani JR, Blau DM, Gurley ES, et al Child deaths caused by Klebsiella pneumoniae in sub-Saharan Africa and south Asia: a secondary analysis of Child Health and Mortality Prevention Surveillance (CHAMPS) data. Lancet Microbe 2024; 5:e131–41. 38218193 10.1016/S2666-5247(23)00290-2 PMC10849973

[18] Mahtab S, Blau DM, Madewell ZJ, et al Post-mortem investigation of deaths due to pneumonia in children aged 1–59 months in sub-Saharan Africa and South Asia from 2016 to 2022: an observational study. Lancet Child Adolesc Health 2024; 8:201–13. 38281495 10.1016/S2352-4642(23)00328-0 PMC10864189

[19] Lam MM, Wick RR, Watts SC, et al A genomic surveillance framework and genotyping tool for Klebsiella pneumoniae and its related species complex. Nat Commun 2021; 12:4188. 34234121 10.1038/s41467-021-24448-3 PMC8263825

[20] Marais G, Moodley C, Claassen-Weitz S, et al Carbapenem-resistant Klebsiella pneumoniae among hospitalized patients in Cape Town, South Africa: molecular epidemiology and characterization. JAC Antimicrob Resist 2024; 6:dlae050. 38529003 10.1093/jacamr/dlae050 PMC10963078

[21] Sands K, Carvalho MJ, Portal E, et al Characterization of antimicrobial-resistant gram-negative bacteria that cause neonatal sepsis in seven low- and middle-income countries. Nat Microbiol 2021; 6:512–23. 33782558 10.1038/s41564-021-00870-7 PMC8007471

[22] Mbelle NM, Feldman C, Sekyere JO, et al Pathogenomics and evolutionary epidemiology of Multi-Drug Resistant Clinical Klebsiella pneumoniae isolated from Pretoria, South Africa. Sci Rep 2020; 10:1232. 31988374 10.1038/s41598-020-58012-8 PMC6985128

[23] Peltier F, Choquet M, Decroix V, et al Characterization of a multidrug-resistant Klebsiella pneumoniae ST607-K25 clone responsible for a nosocomial outbreak in a neonatal intensive care unit. J Med Microbiol 2019; 68:67–76. 30507374 10.1099/jmm.0.000884

[24] World Health Organization . Report signals increasing resistance to antibiotics in bacterial infections in humans and need for better data. Switzerland: World Health Organization, 2022.

[25] Meiring S, Quan V, Mashau R, et al Pathogen aetiology and risk factors for death among neonates with bloodstream infections at lower-tier South African hospitals: a cross-sectional study. Lancet Microbe 2025; 6:100989. 40020700 10.1016/j.lanmic.2024.100989 PMC12062197

[26] Pu D, Zhao J, Chang K, Zhuo X, Cao B. “Superbugs” with hypervirulence and carbapenem resistance in Klebsiella pneumoniae: the rise of such emerging nosocomial pathogens in China. Sci Bull (Beijing) 2023; 68:2658–70. 37821268 10.1016/j.scib.2023.09.040

[27] Silvester R, Madhavan A, Kokkat A, et al Global surveillance of antimicrobial resistance and hypervirulence in Klebsiella pneumoniae from LMICs: an in-silico approach. Sci Total Environ 2022; 802:149859. 34464800 10.1016/j.scitotenv.2021.149859

[28] Stanton TD, Keegan SP, Abdulahi JA, et al Distribution of capsule and O types in Klebsiella pneumoniae causing neonatal sepsis in Africa and South Asia: meta-analysis of genome-predicted serotype Prevalence and potential vaccine coverage. medRxiv 25330253 [Preprint]. June 30, 2025. Available at: 10.1101/2025.06.28.25330253 PMC12810917 41525325

[29] Ramsamy Y, Mlisana KP, Allam M, et al Genomic analysis of carbapenemase-producing extensively drug-resistant Klebsiella pneumoniae isolates reveals the horizontal spread of p18-43_01 plasmid encoding bla (NDM-1) in South Africa. Microorganisms 2020; 8:137. 31963608 10.3390/microorganisms8010137 PMC7023316

[30] Magobo RE, Ismail H, Lowe M, et al Outbreak of NDM-1–and OXA-181–producing klebsiella pneumoniae bloodstream infections in a neonatal unit, South Africa. Emerg Infect Dis 2023; 29:1531. 37486166 10.3201/eid2908.230484 PMC10370860

[31] Agyepong N, Govinden U, Owusu-Ofori A, et al Genomic characterization of multidrug-resistant ESBL-producing Klebsiella pneumoniae isolated from a Ghanaian teaching hospital. Int J Infect Dis 2019; 85:117–23. 31129424 10.1016/j.ijid.2019.05.025

[32] Wysocka M, Zamudio R, Oggioni MR, Gołębiewska J, Dudziak A, Krawczyk B. The new Klebsiellapneumoniae ST152 variants with hypermucoviscous phenotype isolated from renal transplant recipients with asymptomatic bacteriuria-genetic characteristics by WGS. Genes (Basel) 2020; 11:1189. 33066176 10.3390/genes11101189 PMC7601988

[33] Mills RO, Dadzie I, Le-Viet T, et al Genomic diversity and antimicrobial resistance in clinical Klebsiella pneumoniae isolates from tertiary hospitals in Southern Ghana. J Antimicrob Chemother 2024; 79:1529–39. 38751093 10.1093/jac/dkae123 PMC11215549

[34] Salvador-Oke KT, Pitout JDD, Peirano G, et al Molecular epidemiology of carbapenemase-producing Klebsiella pneumoniae in Gauteng South Africa. Sci Rep 2024; 14:27337. 39521758 10.1038/s41598-024-70910-9 PMC11550437

[35] Heinz E, Pearse O, Zuza A, et al Longitudinal analysis within one hospital in sub-Saharan Africa over 20 years reveals repeated replacements of dominant clones of Klebsiella pneumoniae and stresses the importance to include temporal patterns for vaccine design considerations. Genome Med 2024; 16:67. 38711148 10.1186/s13073-024-01342-3 PMC11073982

[36] Olwagen CP, Izu A, Khan S, et al Genomic relatedness of colonizing and invasive disease Klebsiella pneumoniae isolates in South African infants. Sci Rep 2025; 15:8043. 40055469 10.1038/s41598-025-92517-4 PMC11889247

